# Persistent inflammatory state after photoreceptor loss in an animal model of retinal degeneration

**DOI:** 10.1038/srep33356

**Published:** 2016-09-14

**Authors:** Agustina Noailles, Victoria Maneu, Laura Campello, Violeta Gómez-Vicente, Pedro Lax, Nicolás Cuenca

**Affiliations:** 1 Physiology, Genetics and Microbiology, University of Alicante, Alicante, Spain; 2 Optics, Pharmacology and Anatomy, University of Alicante, Alicante, Spain

## Abstract

Microglia act as the resident immune cells of the central nervous system, including the retina. In response to damaging stimuli microglia adopt an activated state, which can progress into a phagocytic phenotype and play a potentially harmful role by eliciting the expression and release of pro-inflammatory cytokines. The aim of the present study was to assess longitudinal changes in microglia during retinal degeneration in the homozygous P23H rat, a model of dominant retinitis pigmentosa. Microglial phenotypes, morphology and density were analyzed by immunohistochemistry, flow cytometry, and cytokine antibody array. In addition, we performed electroretinograms to evaluate the retinal response. In the P23H retina, sclera, choroid and ciliary body, inflammatory cells increased in number compared with the control at all ages analyzed. As the rats became older, a higher number of amoeboid MHC-II^+^ cells were observed in the P23H retina, which correlated with an increase in the expression of pro-inflammatory cytokines. These findings suggest that, in the P23H model, retinal neuroinflammation persists throughout the rat’s life span even after photoreceptor depletion. Therefore, the inclusion of anti-inflammatory drugs at advanced stages of the neurodegenerative process may provide better retinal fitness so the remaining cells could still be used as targets of cellular or gene therapies.

Retinitis pigmentosa (RP) is, from a clinical and genetic point of view, a highly heterogeneous retinal dystrophy characterized by primary degeneration of rod photoreceptors. As RP evolves, only foveal cone photoreceptors remain functional; this is responsible for the characteristic tunnel vision. Cone degeneration, which follows that of the rods, leads to the loss of the central visual field and, eventually, to complete blindness^1^. Over 100 different mutations in the gene encoding rhodopsin (RHO) are associated with 30 to 40% of autosomal dominant cases of RP^2^. One of the mutations is the Pro-23-His substitution, which causes misfolding and retention of rhodopsin in the endoplasmic reticulum^1^,^3^,^4^,^5^. The P23H rat model of RP closely resembles the human disease and has long been considered a valuable tool for the study of retinal dystrophies^3^,^6^,^7^,^8^.

To date, there are no effective treatments for RP, and therapeutic approaches are aimed at halting or slowing down the progression of the disease. Nevertheless, numerous investigations are focused on the development of novel cell and gene therapies, the ultimate goal of which is the functional recovery of the retina. Although in some retinal dystrophies, such as Leber’s congenital amaurosis, gene therapy has initially improved visual acuity, over the long term it has been unable to stop the continuing loss of photoreceptors^9^. This suggests that perhaps other factors, such as the inflammatory state of the tissue, might be involved in the progression of retinal degenerations and should be taken into account for the appropriate design of combined therapies. In this regard, it has been shown that neuroinflammation and reactive gliosis go hand in hand with photoreceptor degeneration in animal models of RP (reviewed in ref. 10). In fact, recent studies place the focus on microglia, demonstrating that these glial cells directly contribute to non-cell-autonomous neuronal loss, as they are responsible for the phagocytosis of living neurons in the stressed brain and retina^11^,^12^.

Microglia represent the resident immune population of the retina and are involved in the maintenance of tissue integrity under physiological conditions, participating in axonal growth, synaptic remodeling and neuronal survival^13^,^14^,^15^. In the absence of a negative stimulus, retinal microglia exhibit a surveillance state, morphologically characterized by numerous branched processes arising from a small, round soma. In response to harmful stimuli, retinal microglia develop an amoeboid reactive form lacking cellular processes and exhibiting macrophage behavior. Active microglia can proliferate, migrate towards the damaged sites and secrete molecules that initiate tissue repair mechanisms, favoring neuroprotection^16^,^17^. However, if activation is excessive or prolonged, the constant secretion of nitric oxide and pro-inflammatory cytokines (e.g. IL-1α, IL-1β, TNF-α, IFN-γ, IL-6) may lead to chronic inflammation and potential pathological side effects, including neuronal apoptosis^18^,^19^,^20^. Active microglia have been described in RP^8^,^12^,^21^,^22^,^23^,^24^ and other retinal neurodegenerative diseases, such as age-related macular degeneration^14^,^25^ or glaucoma^14^,^26^,^27^,^28^,^29^,^30^. In addition to retinal degenerations, some neurodegenerative disorders, such as Parkinson’s or Alzheimer’s disease, have also been linked to microglia activation and high levels of pro-inflammatory molecules^31^,^32^,^33^. These data suggest that it is a common phenomenon in the pathophysiology of neurodegenerations and may influence their progression.

Because the inflammatory response seems to be a critical factor for neuronal survival^11^,^12^, the success of retinal cell or gene therapies might rely, to some degree, upon the favorable conditions of the target tissue, in terms of neuroinflammation. In this context, we have found few studies that investigate how microglia evolve over time in a chronic retinal disease^34^. Therefore, our objective was to determine the onset of microglia activation in an animal model of RP and to characterize the inflammatory profile of the retina, choroid and sclera at early, intermediate and advanced stages of the disease, with the purpose of clarifying whether retinal neuroinflammation persists once the majority of photoreceptors have died. We hope this knowledge will help in the design of more effective therapeutic approaches.

## Results

### The decay of retinal function in the P23H rat correlates with alterations in Iba1-positive cell morphology and surface marker expression

**Figure 1:**
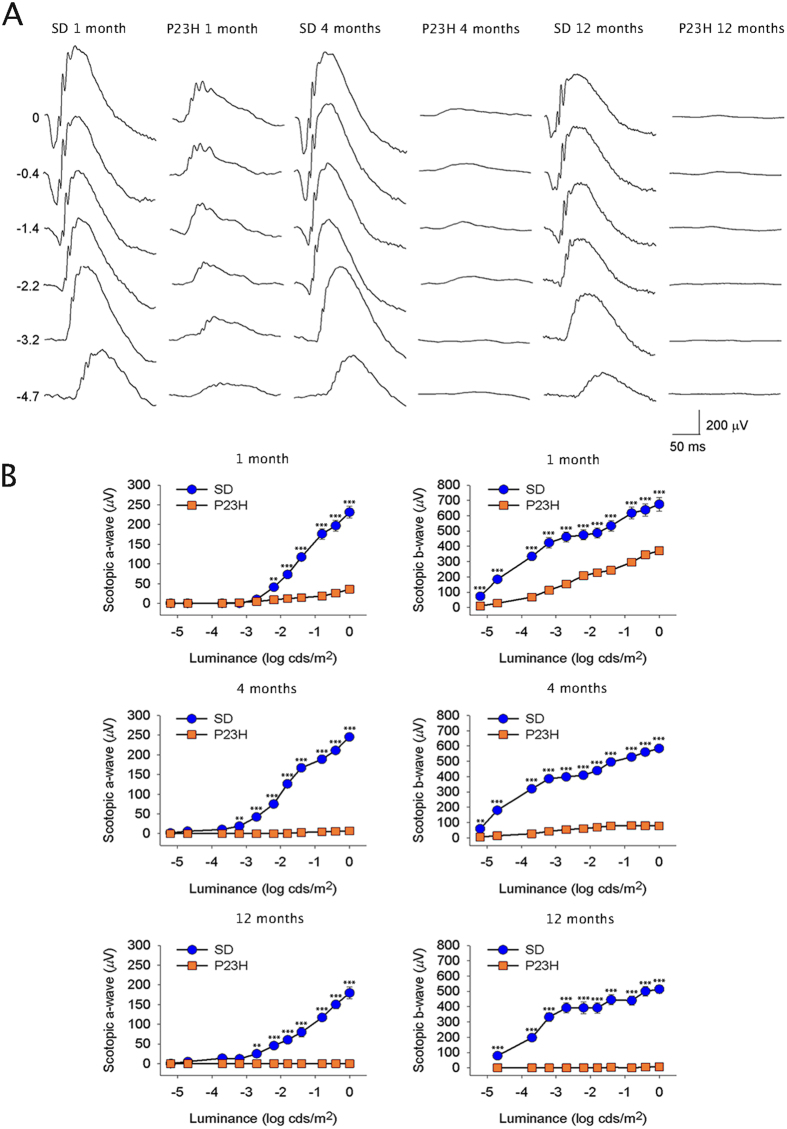
Retinal responsiveness. (**A**) Scotopic ERG waveforms of 1-, 4- and 12-month-old SD and P23H rats. Units on the left of the panel indicate input flash intensities in log cd s/m^2^; (**B**) Luminance-response curves of 1-, 4- and 12-month-old SD (blue/circles) and P23H (orange/squares) rats. Data are represented as mean ± SEM, n = 6.

Scotopic electroretinographic (ERG) responses were recorded from 1-, 4- and 12-month-old Sprague-Dawley (SD; control) and P23H rats, three time points that represent early, intermediate and advanced stages of the disease. As shown in Fig. 1, retinal functionality was maintained over time in SD rats, although it was slightly reduced at 12 months of age. However, a premature drop in the ERG response was observed in 1-month-old P23H rats, where the maximum a- and b-wave amplitudes were diminished by 84% and 45%, respectively, as compared to age-matched SD rats. In a similar manner, 4-month-old P23H animals showed a decrease in the maximum a- and b-wave amplitudes of 97% and 86% respectively, and at 12 months, the decline had already reached levels of 100%.

**Figure 2:**
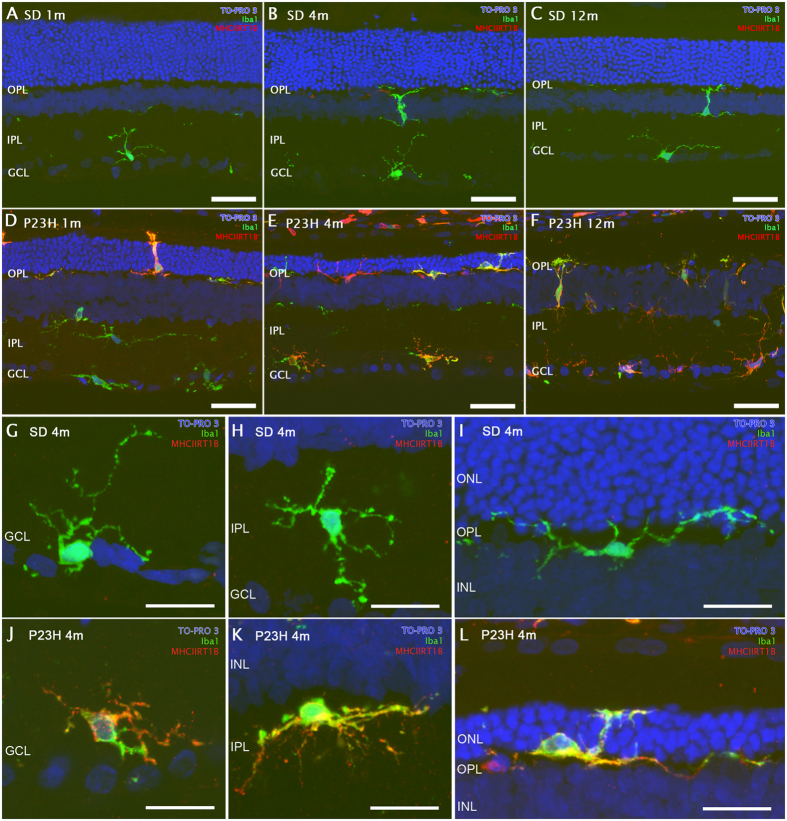
Microglia morphology and surface marker expression. Representative images from vertical sections of 1-, 4- and 12-month-old SD (**A–C**) and P23H (**D–F**) retinas, stained with Iba1 (green) and MHC-II RT1B (red) antibodies. Nuclei were counterstained with TO-PRO-3 iodide (blue). Higher magnification images of 4-month-old SD (**G–I**) and P23H (**J–L**) retinas. All images were acquired in the central retina. GCL, ganglion cell layer; IPL, inner plexiform layer; INL, inner nuclear layer; OPL, outer plexiform layer; ONL, outer nuclear layer. Scale bars 40 μm (**A–F**) and 20 μm (**G–L**).

In P23H rats, the age-dependent loss of retinal responsiveness is associated with a gradual thinning of the photoreceptor cell layer^6^,^7^,^35^ and comes often accompanied by an increase in microglia numbers and changes in their morphology and/or surface marker expression^8^. To look for possible signs of microglia activation, retinal sections from 1-, 4- and 12-month-old SD and P23H rats were immunostained with antibodies against Iba1, constitutively expressed by microglia^36^, and MHC-II, the upregulation of which is a hallmark of reactive microglia^37^ (Fig. 2). In SD rat retinas, Iba1^+^ cells exhibited a ramified morphology, with a tiny soma, little perinuclear cytoplasm and a large number of fine branching processes. This characteristic morphology remained unchanged from 1 to 12 months of age, and the cells displayed little or no immunoreactivity against MHC-II antibodies (Fig. 2A–C). In contrast, changes in Iba1^+^ cells were evident in the P23H retina, starting at 1 month of age. Although some Iba1^+^ cells were observed in 1-month-old retinas with a morphology that was almost normal (Fig. 2D), as the animals aged, their somas became gradually enlarged, presenting a few short, thick primary and terminal processes, and became immunopositive for MHC-II, all of which are characteristic of reactive microglia (Fig. 2E,F). The typical morphological features of resting and reactive microglia can be better appreciated in representative higher magnification images of 4-month-old SD (Fig. 2G–I) and P23H (Fig. 2J–L) retinas. These results evidenced that the decay of retinal function in the P23H rat correlated with changes in Iba1^+^ cell morphology and expression of MHC-II.

### Iba1-positive cell numbers and distribution across retinal layers are altered in the P23H rat from early stages of the disease on

**Figure 3:**
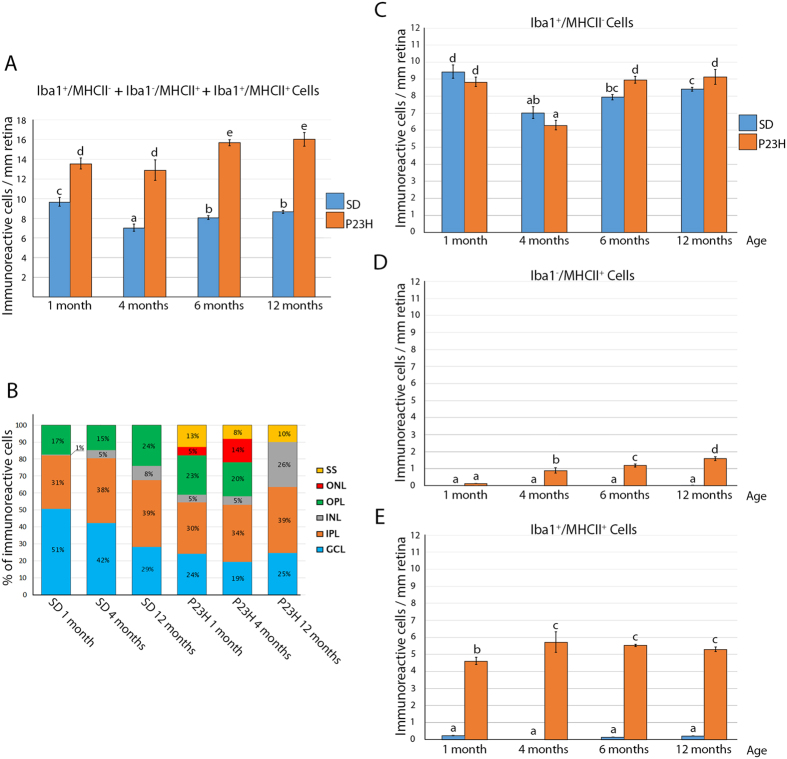
Quantification and spatial distribution of Iba1 and/or MHC-II immunoreactive cells in retinal sections. (**A**) Average number of Iba1^+^ and/or MHC-II^+^ cells per mm of retina; (**B**) Distribution of Iba1^+^ and/or MHC-II^+^ cells across retinal layers. Bars represent the percentage of Iba1^+^ and/or MHC-II^+^ cells in each layer with respect to the total (100%) in the same retinal section. GCL, ganglion cell layer; IPL, inner plexiform layer; INL, inner nuclear layer; OPL, outer plexiform layer; ONL, outer nuclear layer; SS, subretinal space; (**C–E**) Average number of cells expressing Iba1^+^/MHC-II^−^ (**C**), Iba1^−^/MHC-II^+^ (**D**) or Iba1^+^/MHC-II^+^ (**E**) markers. All data are plotted as mean ± SEM, n = 4, ANOVA, Bonferroni *post-hoc*. Different letters indicate statistical significance.

Due to the fact that Iba1^+^ cells in the P23H retina displayed the phenotype of reactive microglia, as confirmed by morphological changes and MHC-II expression, we decided to analyze the effect of RP progression on Iba1^+^ cell numbers and distribution. The total number of cells expressing Iba1, MHC-II or both markers was determined in retinal sections. Immunopositive cells were significantly higher in P23H retinas at all time points, as compared to age-matched SD retinas (140% at 1 month, 184% at 4 months, 194% at 6 months and 185% at 12 months of age) (Fig. 3A). Importantly, while Iba1^+^ cells were barely found in the outer retina of SD rats (Fig. 2A–C), we could observe abundant immunoreactive cells in the outer nuclear layer (ONL) of P23H animals (Fig. 2D,E). Thus, we decided to confirm changes in distribution by quantifying their relative numbers in each retinal layer (Fig. 3B). In SD retinas, we observed slight variations in Iba1^+^ cell numbers that may be attributed to normal aging. Significant differences were detected in the ganglion cell layer (GCL), whereIba1^+^cell density progressively diminished as the animals aged. In the outer plexiform layer (OPL), the percentage of these cells was similar at 1 and 4 months of age, but a significant increase was observed at 12 months (Fig. 3B). In contrast, a profound reorganization of immunoreactive cells took place in P23H retinas. Specifically, the percentage of these cells in the ONL significantly increased between 1 and 4 months of age, but it was not possible to quantify them in 12-month-old retinas because of the complete degeneration of the ONL at these advanced stages of the disease. Also, we noticed a significant increase between 4 and 12 months of age in the INL, which could be attributed, at least in part, to relocation from the degenerated outer retina. In addition, P23H rats exhibited a constant population of Iba1^+^ cells in the subretinal space (SS) at all time points, while these cells were absent in SD animals.

Finally, we performed a phenotypic classification of the cells on the basis of their surface marker expression (Fig. 3C–E). The number of MHCII^+^ cells (either positive or negative for Iba1) was higher in P23H retinas at all time points (Fig. 3D,E). Differences between SD and P23H retinas in the number of Iba1^+^/MHCII^−^ cells were only significant at 6 and 12 months of age (Fig. 3C).

**Figure 4:**
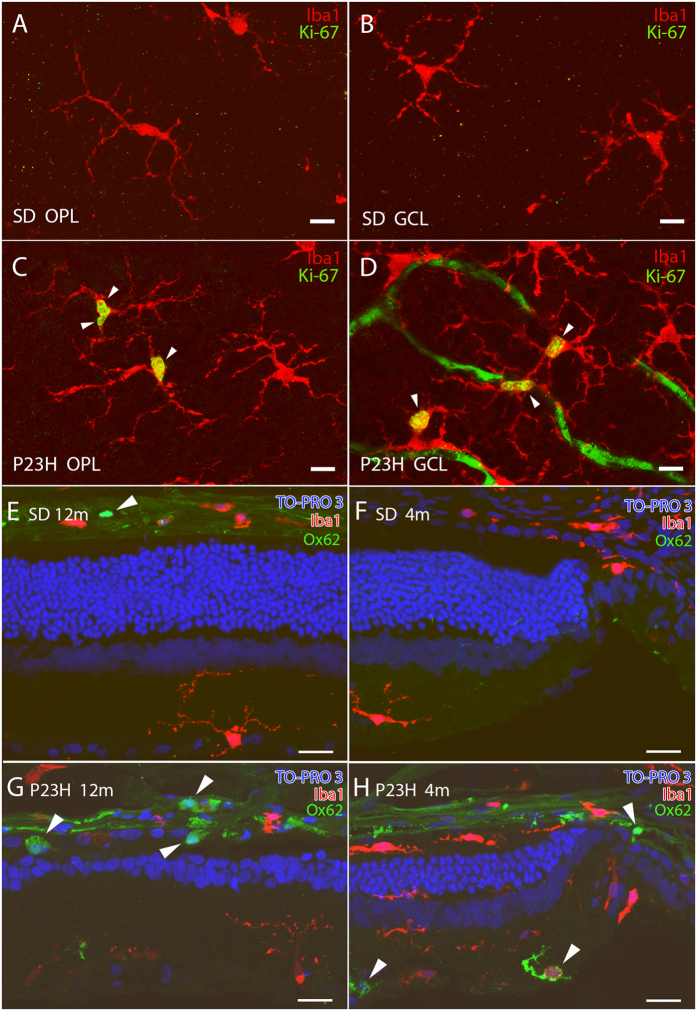
Immunohistochemical detection of proliferative Iba1^+^ and dendritic cells. (**A–D**) Representative images from retinal whole-mounts of 1-month-old SD (**A,B**) and P23H (**C,D**) rats, stained with Ki-67 (green) and Iba1 (red) antibodies. Arrowheads point to Ki-67 immunopositive cells. (**E–H**) Retinal sections of SD (**E,F**) and P23H (**G,H**) rats, immunostained for Iba1 (red) and the dendritic marker Ox-62 (green). Arrowheads indicate immunopositive dendritic cells. OPL, outer plexiform layer; GCL, ganglion cell layer. (**A–D**) Scale bar: 10 μm; (**E–H**) Scale bar: 20 μm.

One potential explanation for the increase in Iba1^+^ cells observed in P23H retinas would be the proliferation of resident microglia. Although under physiological conditions, microglia turnover in the murine retina does not seem to be due to proliferation^38^, the presence of dividing Iba1^+^ cells has been reported in injured retinas^39^. In order to assess this possibility, we performed double immunohistochemical staining with anti-Iba1 and anti-Ki-67 antibodies in 1-month-old whole-mount retinas. Several proliferating Iba1^+^ cells were found in the GCL and OPL of P23H retinas, mainly in the periphery (Fig. 4C,D). We could not observe any double-labeled cells in SD retinas (Fig. 4A,B).

Another possible reason for the increase in the retinal Iba1^+^ population may be attributed to changes in the number of dendritic cells, an inflammatory cell type that also expresses this marker. To assess this, we performed double immunohistochemical staining with anti-Iba1 and anti-integrin alpha E2 (CD103, clone OX-62) antibodies in 1-, 4- and 12-month-old retinal sections. Dendritic cells were found in the choroid and ciliary body of P23H rats at these ages. However, although a few dendritic cells were found in the P23H peripheral retina, close to the ora serrata, we did not find dendritic cells in the rest of the P23H retina (Fig. 4E–H).

Altogether, these results demonstrate that in this model of retinal degeneration, Iba1^+^ cells are activated from early to advanced stages of the disease. The increase in Iba1^+^ cells observed during long-term degeneration in P23H retinas seems to be due, at least in part, to the proliferation of resident microglia.

### A flow cytometric analysis of retinal inflammatory cells further confirms increased numbers in the degenerative retinas of P23H rats

**Figure 5:**
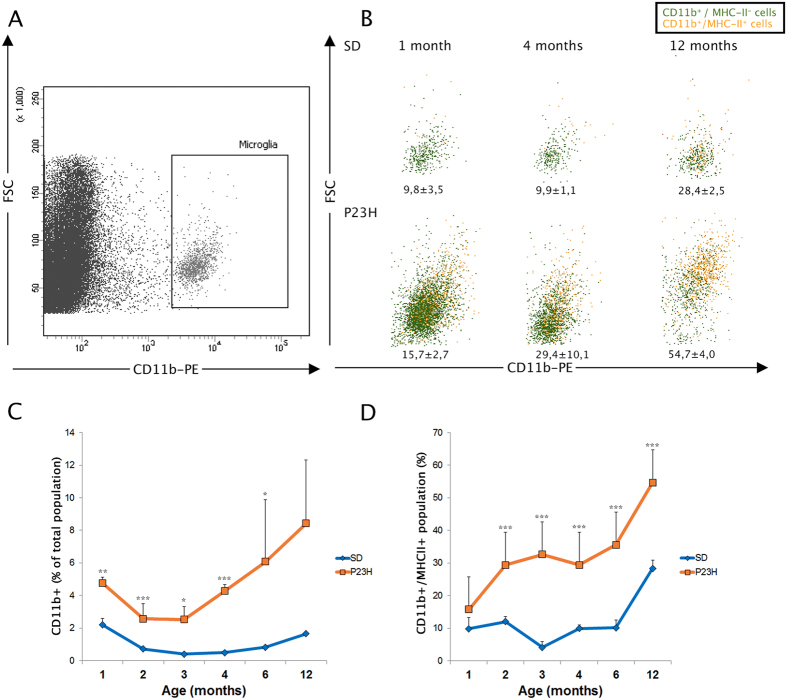
Flow cytometric analysis of retinal inflammatory cells. Disaggregated single retinas were labeled with a cocktail of PE-conjugated anti-CD11b, APC-conjugated anti-MHC class II and FITC-conjugated anti-CD45 antibodies, and analyzed by flow cytometry. (**A**) Representative forward scatter (FSC) *vs*. CD11b dot plot, where CD11b^+^ cells are gated. (**B**) FSC *vs*. CD11b^+^ dot plots, where negative (green) or positive (orange) MHC-II cells are shown. Images correspond to a single experiment representative of at least 3 replicates. 10^6^ events were acquired per sample and 500,000 events are shown. The percentage of MHC-II^+^ cells within the CD11b^+^ population is indicated. (**C**) Percentage of CD11b^+^ cells in the entire retina and (**D**) percentage of MHC-II^+^ cells within the CD11b^+^ population, for SD (diamonds, blue) or P23H (squares, orange) rats at different ages. Data are plotted as mean ± standard deviation. Asterisks indicate significant differences between SD and P23H groups; *P < 0.05, **P < 0.01, ***P < 0.001, Student’s t-test.

Although immunofluorescence is the technique of choice to visualize changes in inflammatory cell morphology and spatial distribution, we sought to analyze retinal inflammatory cells from both a qualitative and quantitative perspective, using an alternative method. Retinal CD11b^+^ cells were identified by flow cytometry (Fig. 5A) and their degree of activation was evaluated by measuring MHC-II (Fig. 5B) and CD45 (not shown) expression. In P23H retinas, the CD11b^+^ population was greater than in age-matched SD rats from 1 to 12 months of age (Fig. 5C), corroborating our immunofluorescence results, i.e. that retinal neuroinflammation begins at early stages of the disease and is amplified at advanced stages, even when no photoreceptors remain in the ONL. A small CD11b^+^/MHC-II^+^ population was observed in the retinas of SD rats up to 6 months of age (Fig. 5D). However, a significantly higher CD11b^+^/MHC-II^+^ population was found in age-matched P23H retinas, which was even greater in 12-month-old animals (Fig. 5D). Double immunostaining against CD11b and CD45 markers evidenced upregulation of CD45 in P23H retinal inflammatory cells, reaching significant levels at 1, 6 and 12 months of age, as compared to age-matched SD retinas (1 month = 1.42-fold increase; 6 months = 1.47-fold increase; 12 months = 2.03-fold increase; data not shown). These results confirmed an activated state of CD11b^+^ cells and increased numbers in P23H retinas.

### Inflammatory cell numbers are increased in the sclera, choroid and ciliary body in P23H rats

Previous studies have shown that when a retinal damage occurs, various cell types expressing microglia/macrophage markers reach the site of injury through the choroid and optic nerve^40^,^41^. For that reason, we analyzed changes in the Iba1^+^/MHCII^+^ cell population of the structures surrounding the retina: the sclera, choroid and ciliary body. To study changes in density or activation of these cells in the sclera and choroid, we obtained mosaics of complete retinal sections and analyzed the fluorescence area in pixels in four representative regions of interest. As we previously demonstrated, retinal degeneration in the P23H rat is not uniform, since the temporal area degenerates at a faster rate than the nasal area^6^,^7^. Therefore, we performed analyses of the choroid and sclera along the nasal temporal axis to know whether there was a correlation between retinal degeneration rate and inflammatory cell density in these areas.

**Figure 6:**
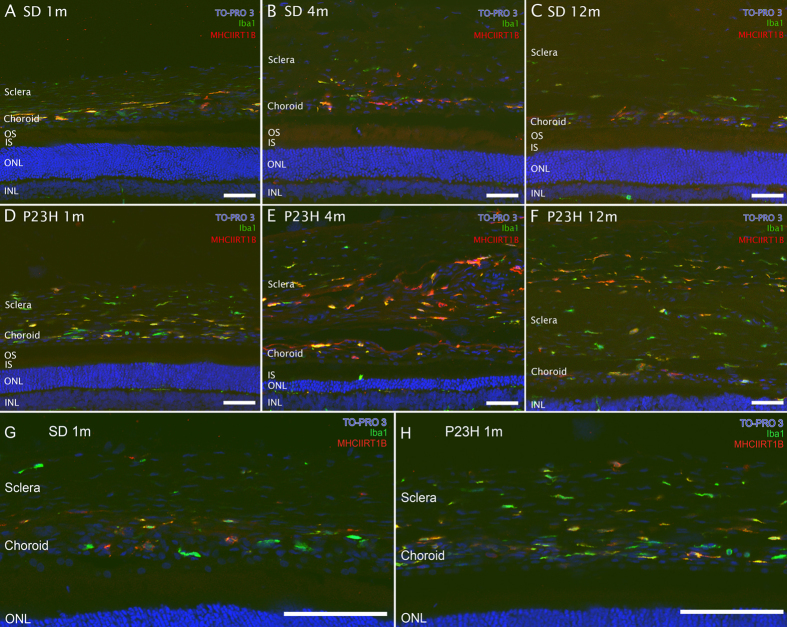
Presence of inflammatory cells in the sclera and choroid. Vertical cryosections of 1-, 4- and 12-month-old SD (**A–C**) and P23H (**D–F**) eyecups, stained with Iba1 (green) and MHC-II RT1B (red). Nuclei were counterstained with TO-PRO-3 iodide (blue). Higher magnification images of 1-month-old SD (**G**) and P23H (**H**) sections. All images were acquired in retinal sections that contained the optic nerve. OS, outer segments; IS, inner segments; ONL, outer nuclear layer. Scale bar 50 μm.

Our results showed that both the sclera and choroid layers had greater immunoreactivity to Iba1 and MHC-II in 1-month-old P23H rats (Fig. 6D,H) than in age-matched SD rats (Fig. 6A,G). Moreover, immunoreactivity to Iba1 and MHC-II further increased in P23H rats (Fig. 6E) as compared to SD rats (Fig. 6B) at 4 months of age. At 12 months, we could not establish clear differences in Iba1 and MHC-II immunoreactivity between P23H (Fig. 6F) and SD (Fig. 6C) groups in the sclera or the choroid.

**Figure 7:**
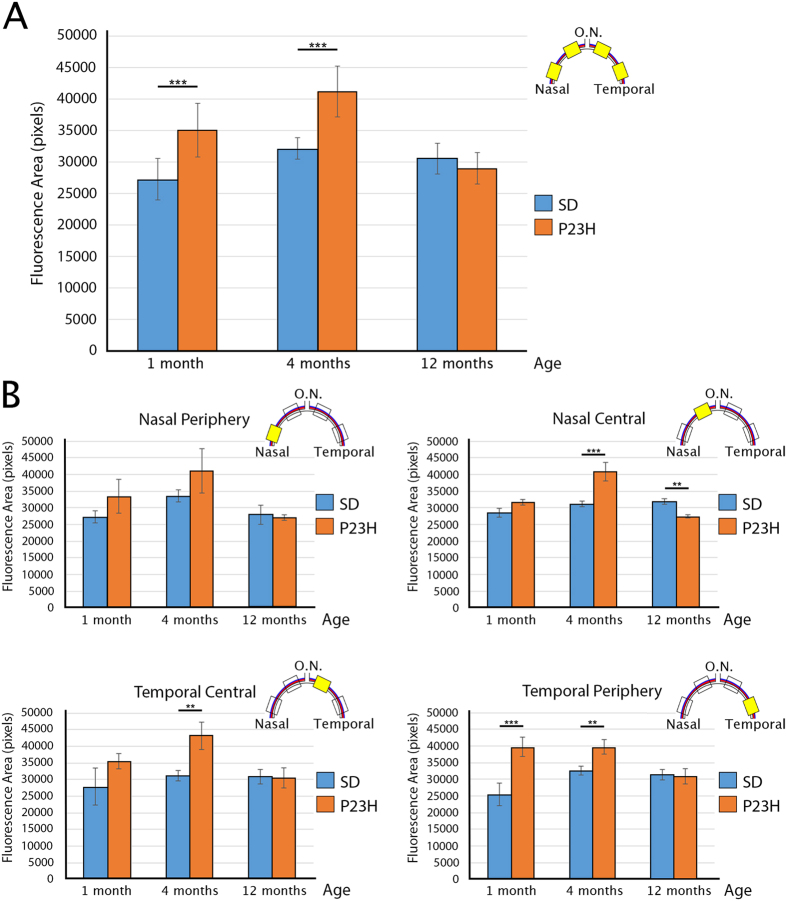
Quantification of inflammatory cells in the sclera and choroid. (**A**) Total fluorescent area (in pixels) of the sclera and choroid in vertical sections of 1-, 4- and 12-month-old SD and P23H retinas. (**B**) Fluorescent area (in pixels) of the sclera and choroid at four different regions of interest (nasal peripheral, nasal central, temporal central and temporal peripheral) of 1-, 4- and 12-month-old SD and P23H retinas. Data are plotted as mean ± standard deviation, n = 4. Asterisks indicate statistical significant differences; **P < 0.01, ***P < 0.001, ANOVA, Bonferroni *post-hoc*.

Quantification of the fluorescent area generated by Iba1 and MHC-II markers in the sclera and choroid in each entire retinal section showed that P23H rats had statistically significant higher values than those observed in SD rats at 1 and 4 months of age (Fig. 7A). The differences in the fluorescent area between SD and P23H rats at 4 months of age remain statistically significant in all the regions tested, except in the nasal peripheral region (Fig. 7B), which coincides the retinal area with the lower degeneration rate.

**Figure 8:**
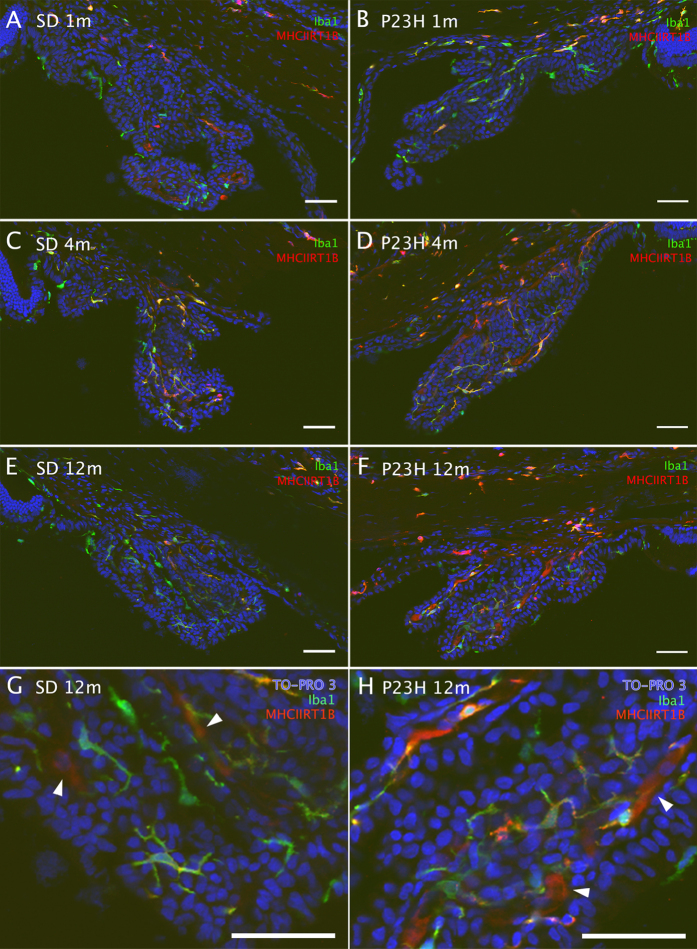
Presence of inflammatory cells in the ciliary body. Representative images from the ciliary body of 1-, 4- and 12-month-old SD (**A,C,E**) and P23H (**B,D,F**) retinas, stained with Iba1 (green) and MHC-II RT1B (red) antibodies. Higher magnification images of 12-month-old SD (**G**) and P23H (**H**) ciliary body. Arrowheads indicatenon-specific immunostaining of blood vessels with the secondary antibody. Scale bar 50 μm.

**Figure 9:**
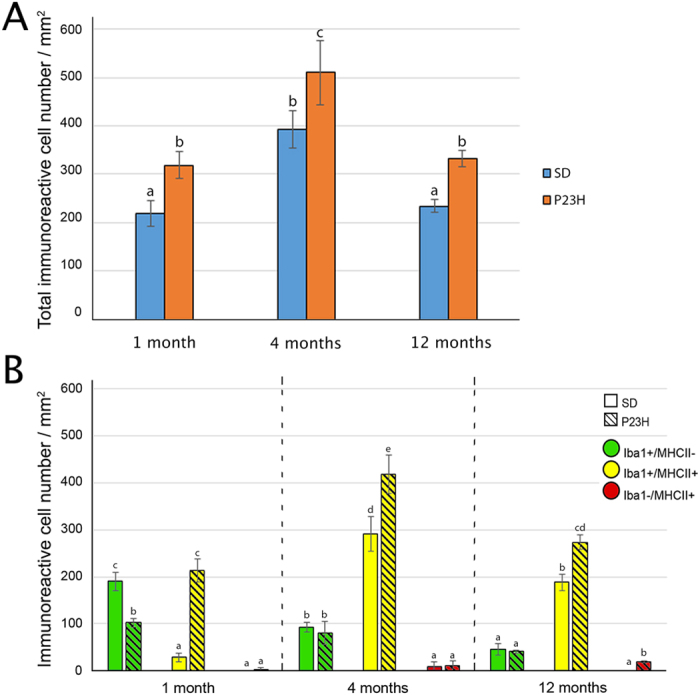
Quantification of inflammatory cells in the ciliary body. (**A**) Density of cells (cells/mm^2^) immunopositive for Iba1 and/or MHC-II markers in the ciliary body of 1-, 4- and 12-month-old SD and P23H rats. (**B**) Number of cells showing the phenotypes Iba1^+^/MHC-II^−^, Iba1^+^/MHC-II^+^ and Iba1^−^/MHC-II^+^ in the ciliary body of 1-, 4- and 12-month-old SD and P23H rats. Data are plotted as mean ± SEM, n = 4, ANOVA, Bonferroni *post-hoc*. Different letters indicate significant differences.

In the ciliary body of SD and P23H rats, Iba1- and/or MHC-II-immunoreactive cells were observed at all ages tested (Fig. 8). At all ages tested, P23H rats showed a greater density of immunopositive cells than that observed in the ciliary body of SD rats at the same age (1.5, 1.3 and 1.4 fold-increase for 1, 4 and 12 months respectively, Fig. 9A). Moreover, MHCII^+^ populations (both Iba1^+^ and Iba1^−^) were higher in P23H than in age-matched SD rats at all ages tested (Fig. 9B), while Iba1^+^/MHCII^−^ populations were higher in SD rats (reaching significance at 1 month of age). In both SD and P23H rats, we observed a significantly decreasing number of Iba1^+^/MHC-II^−^ cells from 1 to 12 months of age. With respect to the Iba1^+^/MHC-II^+^ populations, the number of cells showed a significantly greater peak value at 4 months of age in both animal strains. Regarding the Iba1^−^/MHC-II^+^ cell population, it did not experience any significant changes with age in SD rats, while in P23H rats the number increased as the rat became older, with significant differences between 4 and 12-month-old animals.

Taken altogether, these data suggest that the number of inflammatory cells increases in the sclera, choroid and ciliary body during the first stage of retinal degeneration, and that this increase is uniform over the entire surface of the retina and is therefore independent of the degeneration plaguing the retina in different zones.

### Inflammatory markers persist in advanced stages of retinal degeneration

**Figure 10:**
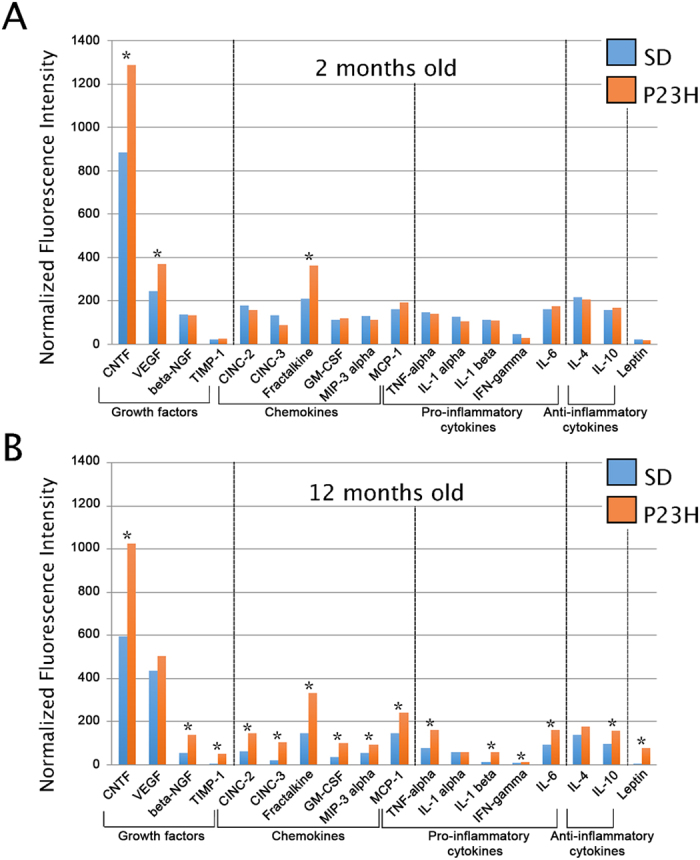
Cytokine-expression profile analysis. Average normalized fluorescence intensity for each pair of cytokine spots in 2 (**A**) and 12 (**B**) month of age SD and P23H retinal extracts. Data are plotted as the mean ± standard deviation, n = 4. Asterisks indicate statistical significant differences; *P < 0.05, Student’s t-test.

To assess whether the evolution of microglial cells in normal and diseased retinas correlated with the inflammatory state of the retinal tissue, we analyzed the expression of pro- and anti- inflammatory molecules in the retinas of 2- and 12-month-old SD and P23H rats, using a cytokine antibody array (Fig. 10). At 2 months of age, P23H retinas showed a significant up-regulation of the chemokine fractalkine (receptor for CX3C chemokine ligand 1; CX3CL1), vascular endothelial growth factor (VEGF) and ciliary neurotrophic factor (CNTF), as compared to age-matched SD retinas (Fig. 10A). At 12 months, P23H rats exhibited a higher concentration of the chemokines: cytokine-induced neutrophil chemoattractant-2 (CINC2), cytokine-induced neutrophil chemoattractant-3 (CINC3), fractalkine (CX3CL1), granulocyte macrophage colony-stimulating factor (GM-CSF), monocyte chemoattractant protein-1 (MCP-1) and macrophage inflammatory protein-3 alpha (MIP-3α). We also found significantly higher values of the pro-inflammatory cytokines: tumor necrosis factor alpha (TNF-α), interleukin-1 beta (IL-1β), interferon gamma (IFN-γ), interleukin-6 (IL-6) and leptin, and the anti-inflammatory cytokine interleukin-10 (IL-10). In addition, at this age, we detected significantly increased levels of growth factors in P23H: nerve growth factor beta (β-NGF), metallopeptidase inhibitor 1 (TIMP-1) and CNTF (Fig. 10B). These results point to a general persistent inflammatory state of the tissue at advanced stages of retinal degeneration, even once the photoreceptors have completely disappeared.

## Discussion

The present study demonstrates the existence of a persistent long-term inflammatory state in the retinal layers, sclera, choroid and ciliary body of a rat model of retinal degeneration. Previous studies have demonstrated the relationship between microglia activation and neurodegenerative diseases, including RP^8^,^42^. However, to our knowledge, there is no previous evidence demonstrating inflammatory conditions at later stages of the degenerative process, which in RP animal models is characterized by the loss of most of the photoreceptors and a profound remodeling of the inner retina. The demonstration of inflammatory conditions in advanced phases of an eye-related neurodegenerative disease could be useful to improve the therapeutic approaches for these pathologies, as well as for other neurodegenerative diseases coursing with neuroinflammation.

In this work, inflammatory cells were identified by their immunoreactivity against two markers expressed by microglia, macrophages and dendritic cells: Iba1 protein^36^ and CD11b integrin^43^. Activation of inflammatory cells was assessed by morphological changes and the expression of MHC class II antigens^37^ and leukocyte common antigen CD45^44^,^45^. We have demonstrated that, while the total number of immunopositive cells for one or more specific markers (Iba1, CD11b and MHC-II) remains fairly constant over time in SD rat retinas, the total number of these cells significantly increases with age in the retina of P23H rats. In these animals, the progressive increase in the number of Iba1^+^/MHC-II^+^ cells was particularly significant, which indicates that activation of inflammatory cells is a retinal tissue response sustained over time. Previous works have shown that microglial cells exposed to pro-inflammatory cytokines derived from Th1 cells, such as IFN-γ, TNF-α and GM-CSF, among others, are stimulated to express MHC-II^46^, which could explain the peak in the expression of MHC-II observed in 12-month-old P23H rats.

We have also identified that the distribution of Iba1 and/or MHC-II positive cells across the retinal layers in P23H rat retinas was different as compared to that in the control rats. In SD rats, immunoreactive cells were located in the inner part of the retinal tissue (GCL and IPL), whereas these cells appeared towards the outer layers of the retinal tissue (OPL and ONL) in P23H rats. This result agrees with previous studies showing the migration of microglial cells through the retina in retinal organotypic cultures^47^, and in animal models of retinal degeneration, such as *rd* mouse^24^ or RCS rats^48^.

Another relevant finding reported here is that P23H rat retinas showed a population of Iba1^−^/MHC-II^+^ cells that increased in number with the progression of the disease. In this regard, recent work by our research group evidenced the presence of a small group of MHC-II^+^ cells that were not labeled by anti-Iba1 antibody in P23H rat retinas^8^, which is noteworthy since the Iba1 protein is expressed by microglia, macrophages and dendritic cells. Moreover, it is one of the most important molecules in the motile properties of these cells, due to its participation in the membrane ruffling processes^49^,^50^. The existence of these Iba1^−^/MHC-II^+^ cells can be explained if we consider that the expression pattern of each cell may vary depending on its origin. It may be that these Iba1^−^/MHC-II^+^ cells are migrating cells that travel to the retinal tissue in response to negative stimuli. It should also be considered that Iba1^−^/MHC-II^+^ cells may be down-regulating the expression of Iba1, as a consequence of the nature or duration of the activating signals. Thus, this novel expression pattern may correspond to an intermediate activation state between resting and reactive microglial states. Whatever the answer, the presence of this microglial population characteristically occurs in the P23H rat, and it presumably plays a specific role in the course of the disease.

This work provides conclusive evidence that the number of Iba1 and MHC-II positive cells progressively increases in P23H rat retinas. This effect can be attributed to a recruitment of immune cells (macrophages or dendritic cells) to the retina. A previous work on light-induced retinal damage showed that blood-borne macrophages enter the retina via the optic nerve and ciliary body and migrate to the injured retinal area^41^. These studies also found that in dark control retinas, microglial cells and macrophages were mainly located within choroidal and conjunctival blood vessels, and in connective tissue around the optic nerve. In this work, we have shown that the sclera and choroid of SD rats is immunoreactive to Iba1 and MHC-II, but at levels lower than that observed in the age-matched P23H rat retinas prior to 4 months of age. This could be due to the arrival of circulating monocytes/macrophages to the retina during the early stages of the degenerative process. At later stages of retinal degeneration (12 months of age), we did not observe any significant differences in immunoreactivity to Iba1 and MHC-II markers in choroid and sclera between SD and P23H rats. These results are in agreement with previous studies which showed that, in RP, photoreceptor cell loss is correlated with subsequent deterioration of the retinal capillary network^51^ and lower levels of retinal blood flow^52^. P23H rats exhibit a poor capillary network, with clear signs of degeneration at 4 months of age^6^. Accordingly, the degenerative process may reduce the affluence of macrophage-like cells to the retina via the choroid during later stages of the disease. Also, in the ciliary body, we appreciated a greater total number of immunopositive cells in P23H rats at all ages tested, with a significant increase in the amount of Iba1^−^/MHC-II^+^ cells. The ciliary body is one of the main pathways through which inflammatory cells can reach the retina^41^, and it is assumed that the macrophage-like cells which cannot do it through the choroid at 12 months of age (because of its great deterioration), do reach the retina directly via the ciliary body and optic nerve. It is therefore likely that these inflammatory cells which colonize the ciliary body are ready to enter the retina and sustain the inflammatory conditions. In order to detect the possible arrival of dendritic cells to the damaged retina, we have assessed the presence of dendritic cells in the retina through immunolabeling with the anti-integrin alpha E2 (clone OX62) antibody. We found barely any dendritic cells in the retina of P23H rats, although a scarce amount of cells was observed close to the ora serrata. However, they were found abundantly in the choroid and ciliary body. These results lead us to conclude that the majority of the retinal immunopositive Iba1^+^/MHC-II^+^ cells in the P23H rats are not dendritic cells.

Age-related increases in Iba1 and MHC-II immunopositive cells in P23H rat retinas could also be caused by the proliferation of resident microglia. To elucidate this hypothesis, we used double immunolabeling with Ki67 and Iba1 antibodies. We detected proliferative Iba1^+^ in P23H rat retinas, but not in the healthy control rats, which suggests that the Iba1 immunopositive cell population in P23H rats increases, at least in part, due to the resident microglia proliferation. All these results considered together lead us to conclude that there are at least two mechanisms that underlie age-related increases in Iba1^+^ cells in P23H rat retinas: the recruitment of immune cells to the retina and the proliferation of resident microglia. We suggest that the increase in the Iba1-positive cells observed in the inner layers of the retina is primarily due to proliferative mechanisms, while the increase in the Iba1^+^ cells detected in the outer retinal layers is mainly due to the arrival of immune cells.

Retinal degeneration in P23H rats was associated with changes in the expression of pro- and anti- inflammatory molecules. In the early stages of the degenerative process (2 months of age), we observed significant increases in the expression levels of fractalkine, VEGF and CNTF in P23H rat retinas. We suggest that these three cytokines may mediate the initial response to the photoreceptor cell death and promote the ensuing massive affluence of pro-inflammatory cells and molecules. Prior studies in fractalkine knockout mice showed that this cytokine is able to control microglia migration in the retina^53^. In this sense, the soluble form of fractalkine released from photoreceptors may function as a chemotactic factor, triggering the activation and migration of retinal microglia^54^. In the retinal tissue, various cell types have been described as producing VEGF, among them, the retinal pigment epithelium, Müller cells, pericytes and astrocytes^55^. VEGF is a proangiogenic molecule that promotes vascular permeability^55^. Previous investigations on a model of traumatic brain injury suggest that it is implicated in the recruitment and activation of microglial cells^56^. In animal models of retinal laser injury, an accumulation of microglial cells was observed at the site of damage, accompanied by a subsequent increase in various growth factors and cytokines, such as VEGF, TNF-α and IL-6, among others^57^. CNTF is one of the most frequently studied neurotrophic factors in retinal degenerative diseases, and it has been hypothesized that this molecule is released from damaged cells under pathological condition^58^, as well as from glial cells^59^.

During the late stages of the degenerative process, at 12 months of age, the inflammatory profile in P23H rat retinas was dramatically increased. At this point, we observed increased expression levels of pro-inflammatory molecules, such as TNF-α, IFN-γ, IL-1β and IL-6^57^,^60^,^61^ and chemoattractant molecules, such as CINC-2, CINC-3 and MCP-1. Under these conditions, certain factors, such as CNTF^62^, and β-NGF^63^ were also up-regulated. These neuroprotective factors can also act as chemoattractant signals for microglial/macrophage cells^64^,^65^, demonstrating that one factor could be intrinsically neuroprotective, although its indirect actions could trigger inflammation responses. At the same time, we were also able to detect an increased expression of the neuroprotective factors GM-CSF^66^ and IL-10^67^. This is not surprising, as in a scenario of advanced tissue degeneration, we expect to find a high level of cell death and a mixed pool of pro- and anti-inflammatory cytokines. Any inflammatory process involves an imbalance between pro- and anti- inflammatory cytokines in favor of the former group, with little or no chance of success for the latter. Previous works have shown this to occur in this context and also in the context of neurodegenerative diseases, such as Alzheimer’s disease^10^,^68^,^69^.

All these results are consistent with the existence of a persistent inflammatory state in the P23H rat retina. It is interesting to note that in RP, despite the loss of a large proportion of photoreceptor cells, the retinal tissue continues to present a strong inflammatory condition. Our findings are in accordance with many preceding works in animal models of RP, such as *rd*10 mice and RCS rats, which showed increased pro-inflammatory cytokines and microglia activation in the early stages of retinal degeneration^48^,^70^. Here, we provide new information about how the retinal tissue inflammatory response evolves with age in RP. In this way, we demonstrate that (1) in an early phase of retinal degeneration, the activating microglial signal consisted mainly of VEGF, CNTF and fractalkine; (2) with age, Iba1^+^ cells of P23H rats exhibited an intense variation, not only in the phenotypes expressed, but also in their distribution and numbers through the retinal layers; (3) in late stages of the disease, the amount of pro-inflammatory cytokines notably increased in comparison to healthy retinas; (4) the inflammatory response was also present in the choroid and ciliary body, which may act as a source of pro-inflammatory cells and molecules; and (5) the peak of pro-inflammatory cells in the choroid and ciliary body of P23H rats was observed at a mid-stage of the degenerative process (4 months).

In conclusion, here we provide a new global view of the retinal microglial inflammatory response that is important to take into account for future therapies. The microglia shift to a permanently activated state can trigger a long-term response to the disease that may determine the success or failure of a potential therapy. The maintenance of a healthier condition in the retinal tissue would be important for all the cells in the retina, especially for cones in advanced stages of degeneration, as they survive longer than rods. In this sense, the use of anti-inflammatory drugs, even in late stages of the neurodegenerative process, could preserve the retina in better condition for stem cell transplantation, gene therapy or artificial vision. Likewise, these results maybe important in terms of gaining a better understanding of the mechanisms involved in other neurodegenerative diseases, such as Parkinson’s or Alzheimer’s disease, where similar long-term inflammatory responses can take place.

## Methods

### Animals

Homozygous P23H line 3 rats, kindly provided by Matthew LaVail (UCSF School of Medicine; www.ucsfeye.net/mlavailRDratmodels.shtml), and Sprague-Dawley (SD) rats (Harlan Laboratories, Barcelona, Spain) were used in this study. Animals were housed under controlled photoperiod (12 h light/12 h dark), temperature (23 °C ± 1 °C) and humidity (55–60%) conditions. Food and water were available *ad libitum*. Procedures were carried out under the project license UA-2013-07-22, approved by the Ethics Committee for Animal Experimentation at the University of Alicante. Animals were handled in accordance with current regulations for the use of laboratory animals (NIH, ARVO and European Directive 2010/63/UE), in order to comply with the 3Rs.

### Immunohistochemistry

Histological studies were performed at 1, 4, 6 and 12 months of age. Animals were sacrificed in the morning by a lethal dose of pentobarbital (100 mg/kg, i.p.). After marking the dorsal margin of the sclerocorneal limbus with a suture, the right eyes were enucleated and fixed in 4% (w/v) paraformaldehyde for 1 h at room temperature. Next, they were washed with 0.1 M phosphate buffer with a pH of 7.4 (PB) and sequentially cryoprotected in 15, 20 and 30% (w/v) sucrose. The cornea, lens and vitreous body were removed, the eyecups were embedded in Tissue-Tek OCT (Sakura Finetek, Zoeterwouden, Netherlands) and they were then frozen in liquid N_2_. Sixteen-micrometer-thick sections were obtained on the nasal temporal axis using a Leica CM 1900 cryostat (Leica Microsystems, Wetzlar, Germany). Sections were mounted on slides (Superfrost Plus; Menzel GmbH and Co. KG, Braunschweig, Germany) and washed several times prior to incubation with blocking solution (PB containing 10% (v/v) donkey normal serum and 0.5% (v/v) triton X-100) for 1 h. Then, sections were incubated overnight with the primary antibodies: polyclonal rabbit anti-ionized calcium binding adapter molecule 1 (Iba1, 1:1000; Wako Chemicals, Richmond, VA, USA) and monoclonal mouse anti-major histocompatibility complex (MHC) class II RT1B (clone OX-6, 1:200; AbD Serotec, Kidlington, UK) or monoclonal mouse anti-integrin alpha E2 (CD103 clone OX-62, 1:50; Abcam, Cambridge UK). To assess the presence of proliferative microglia, 1-month-old SD and P23H eyes (n = 4) were fixed as mentioned above. The retinas were then whole-mounted and double-labeled with monoclonal mouse anti-Ki-67 (clone B56, 1:10; BD Biosciences, San Diego, CA, USA) and anti-Iba1. The secondary antibodies were Alexa Fluor^®^ 555/488 anti-mouse or anti-rabbit IgG (1:100; Molecular Probes, Eugene, OR, USA). Photographs were obtained under a Leica TCS SP2 confocal laser-scanning microscope (Leica Microsystems). Images from SD and P23H sections were processed in parallel, using Adobe Photoshop 10 software (Adobe Systems Inc., San Jose, CA, USA).

### Cell counting

To quantify positively immunostained cells, three non-consecutive retinal sections per animal and four animals per age and genotype were examined. Only retinal sections containing the optic nerve were used for quantification, to ensure that the same location was examined in each animal. The total number of cells expressing one or both markers (Iba1^+^/MHC-II^**−**^, Iba1^**−**^/MHC-II^+^ and Iba1^+^/MHC-II^+^) was scored using a Leica DMR fluorescent microscope (Leica Microsystems) under 63X magnification. Data were expressed with reference to the length of each section (in mm), as measured with ImageJ software^71^. In parallel, the percentage of microglia located in each retinal layer (as referred to total microglia (100%) in the same section) was analyzed under 40X magnification.

Additionally, we studied changes in inflammatory cell density and activation in the sclera, choroid and ciliary body in confocal images acquired under 20X magnification. Images of the sclera and choroid were taken at four representative retinal regions: two in the periphery (0.45 mm from the ciliary body on both the nasal and temporal sides) and two in the center (0.45 mm from the optic disc, on both sides). In each image, we established a rectangular region (0.227 mm^2^) for the subsequent measurement of the fluorescence area (in pixels) with ImageJ software.

Statistical analyses were performed using SPSS 20.0 software (IBM, Armonk, NY, USA). A one-way ANOVA was used to compare the mean number of total microglia, and a two-way ANOVA to compare the number of cells corresponding to each phenotype (Iba1^+^/MHC-II^−^, Iba1^−^/MHC-II^+^ and Iba1^+^/MHC-II^+^). When a 0.05 level of significance was found, *post-hoc* pairwise comparisons using Bonferroni’s test were performed. Normal distributions and homogeneity of variance were found for all analyzed categories. Values of P < 0.05 were considered to be statistically significant. Data are plotted as the mean ± standard error of the mean (SEM).

### Flow Cytometry

The left eyes from 1- (SD n = 3; P23H n = 4), 2- (SD n = 4; P23H n = 6), 3- (SD n = 4; P23H n = 6), 4- (SD n = 7; P23H n = 5), 6- (SD n = 4; P23H n = 6) and 12-month-old (SD n = 2; P23H n = 7) rats were enucleated. The retinas were then dissected out, placed in 1 ml of phosphate-buffered saline (PBS) with a pH of 7.4, and disaggregated by gently pipetting up and down through a wide bore pipette tip. This cell suspension was filtered through a 30-μm strainer (BD Biosciences, San Diego, CA, USA) to prevent cell clumps and was triple-stained with a cocktail of antibodies: PE-conjugated anti-rat CD11b/c (clone OX42, eBioscience, San Diego, CA, USA), APC-conjugated anti-rat MHC class II (clone M5/114.15.2; Milteny Biotec, Bergish Gladbach, Germany) and FITC-conjugated anti-rat CD45 (eBioscience). Each retina was examined separately in an LSRFortessa cytometer (BD Biosciences), and a total 10^6^ events were acquired per retina. Data were analyzed with FACSDiva software (BD Biosciences).

A Student’s t-test was performed to compare values between SD and P23H retinas. Results are expressed as the mean ± standard deviation and values of P < 0.05 were considered to be statistically significant.

### Cytokine Array

To determine the expression of inflammatory markers, a commercial rat cytokine antibody array was used (RayBio^®^ rat cytokine antibody array G-series 1; Cat#AAR-CYT-G1-8; RayBiotech, Norcross, GA, USA). Four animals per age and genotype were used and the analysis was performed twice. Protein extracts were obtained by homogenization of 2- and 12-month-old retinas in lysis buffer. Protein concentration was determined in the lysates with the Bio-Rad protein assay (Bio-Rad, Hercules, CA, USA), using bovine serum albumin as a standard. The sample concentration was then adjusted to 1 μg/μl with blocking buffer and 100 μl were loaded per well. Samples were incubated on the microarray chip overnight at 4 °C, with gentle agitation. After washing, the slide was processed strictly following the manufacturer’s protocol and finally scanned at 532 nm. Cytokine expression levels were obtained by measuring the fluorescence intensity of each spot on the microarray chip with GenePix^®^ Pro 6.0 software (Axon Instruments Inc., Union City, CA, USA) and subtracting the background. The values were then normalized to the positive controls included on the chip. For a single protein, any ≥1.5-fold increase or ≤0.65-fold decrease in intensity between groups was considered a significant difference in expression, provided that both sets of signals were well above the background.

A Student’s t-test was used to detect statistical differences between SD and P23H retinas. Values of P < 0.05 were considered to be statistically significant. Results are represented as the mean ± standard deviation.

### Electroretinograms

Scotopic electroretinograms were performed at 1, 4 and 12 months of age. Six animals per age and genotype were registered. Following overnight dark adaptation, animals were anesthetized by an intraperitoneal injection of ketamine (100 mg/kg) and xylazine (4 mg/kg) and maintained on a heating pad at 38 °C. Pupils were dilated by topical administration of 1% tropicamide (Alcon Cusí, Barcelona, Spain). A drop of Viscotears 0.2% polyacrylic acid carbomer (Novartis, Barcelona, Spain) was infused on the cornea to prevent dehydration and to allow electrical contact with the recording electrodes (DTL fiber with an X-Static silver-coated nylon conductive strand; Sauquoit Industries, Scranton, PA, USA). A 25-gauge platinum needle, inserted under the scalp between the eyes, was used as the reference electrode. A gold electrode was placed in the mouth and acted as ground. Animals were placed in a Faraday cage and recordings were performed in absolute darkness. Scotopic flash-induced ERG responses were recorded from both eyes in response to light stimuli produced by a Ganzfeld stimulator. Light stimuli were presented for 10 ms at 11 different and increasing luminances, ranging from −5.2 to 0 log cd s/m^2^. Three to ten consecutive recordings were averaged for each light presentation. The interval between stimuli was 10 s for dim flashes (−5.2 to −1.4 log cd s/m^2^) and up to 20 s for the highest luminances (−0.8 to 0 log cd s/m^2^). ERG signals were amplified and band-pass filtered (1–1000 Hz, without notch filtering) using a DAM50 data acquisition board (World Precision Instruments, Aston, UK). Stimuli presentation and data acquisition (4 kHz) were performed using a PowerLab system (AD Instruments, Oxfordshire, UK). Recordings were analyzed off-line. The amplitude of the a-wave was measured from the baseline. The amplitude of the b-wave was determined from the trough of the a-wave to the peak of the b-wave.

Statistical analysis was performed with SPSS 20.0 software (IBM). A MANOVA was used to evaluate the effects of age progression on ERG responses. When a 0.05 level of significance was observed, *post-hoc* pairwise comparisons using Bonferroni’s test were carried out. Normal distributions and homogeneity of variance were found for all analyzed categories. Values of P < 0.05 were considered to be statistically significant. Data were plotted as the mean ± SEM.

## Additional Information

**How to cite this article**: Noailles, A. *et al*. Persistent inflammatory state after photoreceptor loss in an animal model of retinal degeneration. *Sci. Rep*. **6**, 33356; doi: 10.1038/srep33356 (2016).
